# Supplementary material for: Revealing isoelectronic size conversion dynamics of metal nanoclusters by a noncrystallization approach
Source: Nat Commun. 2018 May 17;9:1979. doi: 10.1038/s41467-018-04410-6 (PMC5958061; doi:10.1038/s41467-018-04410-6)
Supplement: Supplementary file 1 — Supplementary Information [file 41467_2018_4410_MOESM1_ESM.pdf]

<u>Supplementary Information for</u>

Revealing Isoelectronic Size Conversion Dynamics of Metal Nanoclusters by a Noncrystallization Approach

Yao et al.

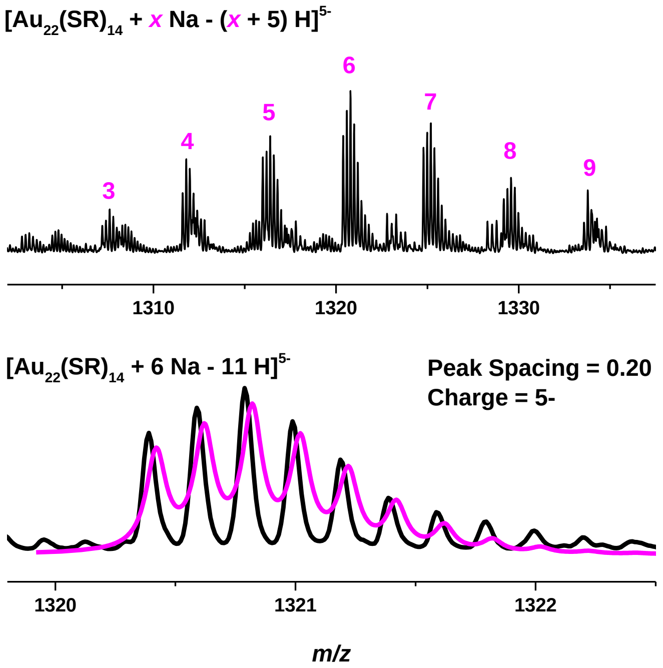

Supplementary Figure 1. Electrospray ionization mass spectrum (top) and isotope patterns (bottom) of fragment $[Au_{22}(SR)_{14}]^0$ (SR = thiolate ligand) indicated as asterisk peaks in Fig. 1d. The magenta line is simulated isotope pattern of $[Au_{22}(SR)_{14} + 6\ Na – 11\ H]^{5-}$.

Supplementary Note 1. Fragility of as-obtained $[Au_{23}(p\text{-MBA})_{16}]^-$

To rule out the interference from fragments in electrospray ionization mass spectrometry (ESI-MS) analysis, we attempted to elude the fragility of $[Au_{23}(p\text{-MBA})_{16}]^-$, where *p*-MBA is *para*-mercaptobenzoic acid, in a semi-aqueous medium by phase-transferring Au NCs into an organic solvent. This phase-transfer was made possible by an ion-pairing reaction between the end carboxylic group of *p*-MBA and the incoming hydrophobic cation (e.g., cetyltrimethylammonium or CTA^+ for short)[1]. As shown in the insets of Supplementary Fig. 2a and 3a, the reaction of $[Au_{23}(p\text{-MBA})_{16}]^-$ with CTACl or CTABr could completely shuttle the resultant $[Au_{23}(p\text{-MBA})_{16}@x\text{CTA}]^-$ NCs (*x* denotes the number of CTA^+ associated with each Au cluster) from aqueous phase into organic phase (e.g., a mixture of toluene and ethanol). The phase-transferred $[Au_{23}(p\text{-MBA})_{16}@x\text{CTA}]^-$ NCs show similar absorption features as those of water-soluble $[Au_{23}(p\text{-MBA})_{16}]^-$ (Supplementary Fig. 2a and 3a). More importantly, all peaks observed in the ESI mass spectra of phase-transferred Au NCs (Supplementary Fig. 2b and 3b) could be attributed to $[Au_{23}(p\text{-MBA})_{16}]^-$ associated with varied number of CTA^+, and no signals for fragment $[Au_{22}(p\text{-MBA})_{14}]^0$ were observed. UV-vis absorption and ESI-MS analyses on the phase-transferred Au_{23} NCs corroborate the molecular purity of as-prepared $[Au_{23}(SR)_{16}]^-$ NCs.

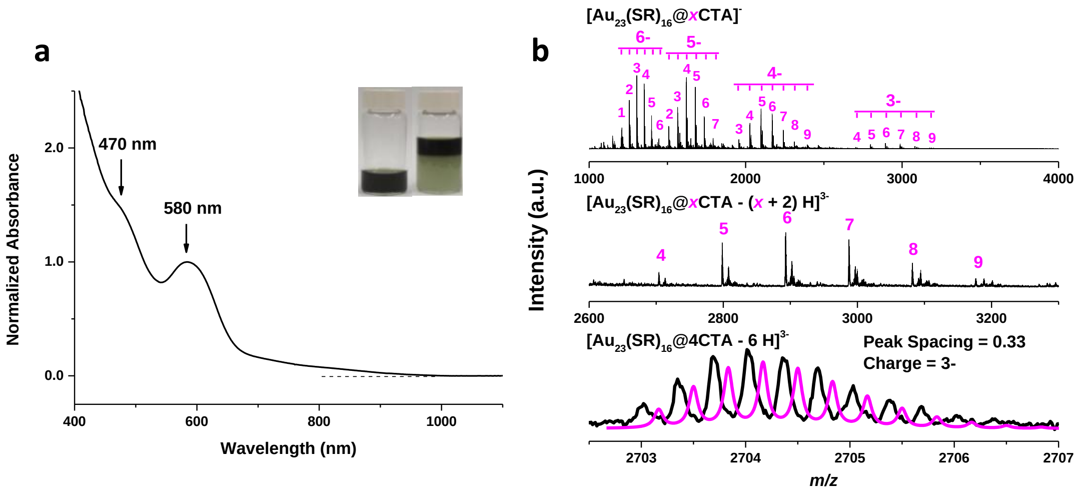

Supplementary Figure 2. (**a**) Ultraviolet-visible absorption and (**b**) electrospray ionization mass spectra of phase-transferred $[Au_{23}(p\text{-MBA})_{16}@x\text{CTA}]^-$ nanoclusters (*p*-MBA = *para*-mercaptobenzoic acid; and CTA = cetyltrimethylammonium) obtained by reacting $[Au_{23}(p\text{-MBA})_{16}]^-$ with CTACl. Insets in (**a**) are digital photos of Au_{23} nanoclusters before (left) and after (right) phase-transfer. The zero absorbance is indicated by the dotted line in (**a**). The *x* values in each ion are shown above the corresponding peaks in top and middle spectra of (**b**), and the magenta line in bottom spectrum of (**b**) is the simulated isotope pattern of $[Au_{23}(p\text{-MBA})_{16}@4\text{CTA} – 6\ H]^{3-}$.

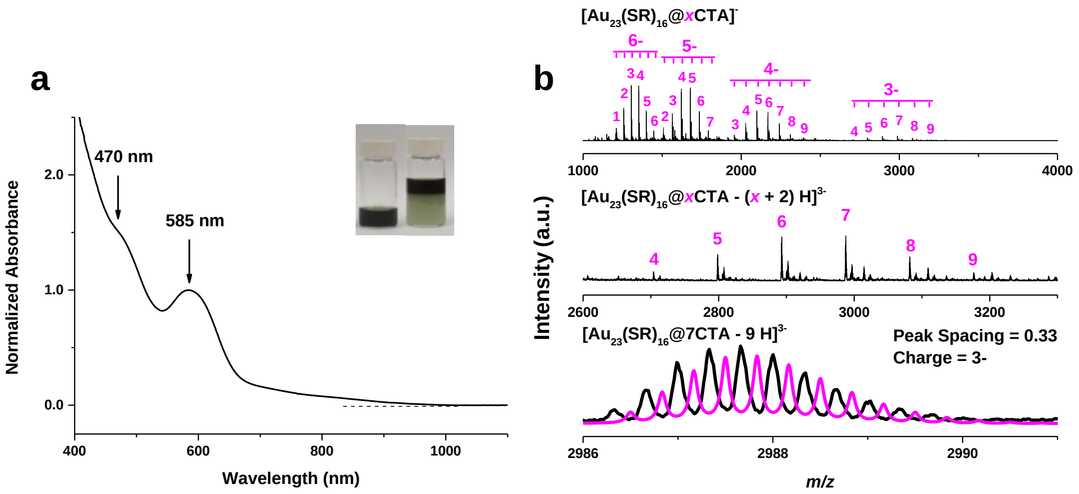

Supplementary Figure 3. (**a**) Ultraviolet-visible absorption and (**b**) electrospray ionization mass spectra of phase-transferred $[Au_{23}(p\text{-MBA})_{16}@x\text{CTA}]^{-}$ nanoclusters (*p*-MBA = *para*-mercaptobenzoic acid; and CTA = cetyltrimethylammonium) obtained by reacting $[Au_{23}(p\text{-MBA})_{16}]^{-}$ with CTABr. Insets in (**a**) are digital photos of Au_{23} nanoclusters before (left) and after (right) phase-transfer. The zero absorbance is indicated by the dotted line in (**a**). The *x* values in each ion are shown above the corresponding peaks in top and middle spectra of (**b**), and the magenta line in bottom spectrum of (**b**) is the simulated isotope pattern of $[Au_{23}(p\text{-MBA})_{16}@7\text{CTA} - 9\ \text{H}]^{3-}$.

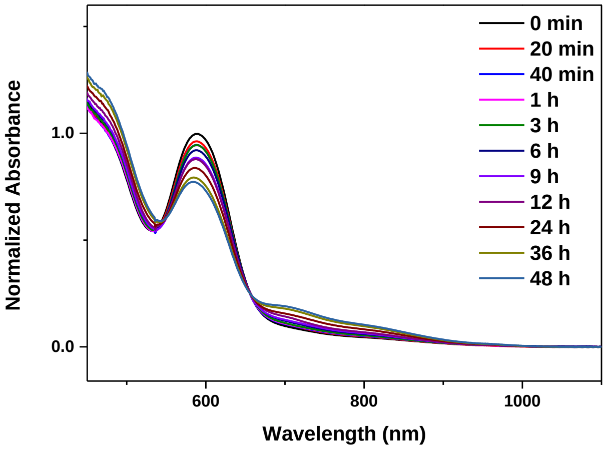

Supplementary Figure 4. Time-evolution ultraviolet-visible absorption spectra of $[Au_{23}(p\text{-MBA})_{16}]^-$ (*p*-MBA = *para*-mercaptobenzoic acid) incubated in water/ethanol (6/4, Vol/Vol). All absorption spectra are normalized to optical density at 589 nm at $t = 0$.

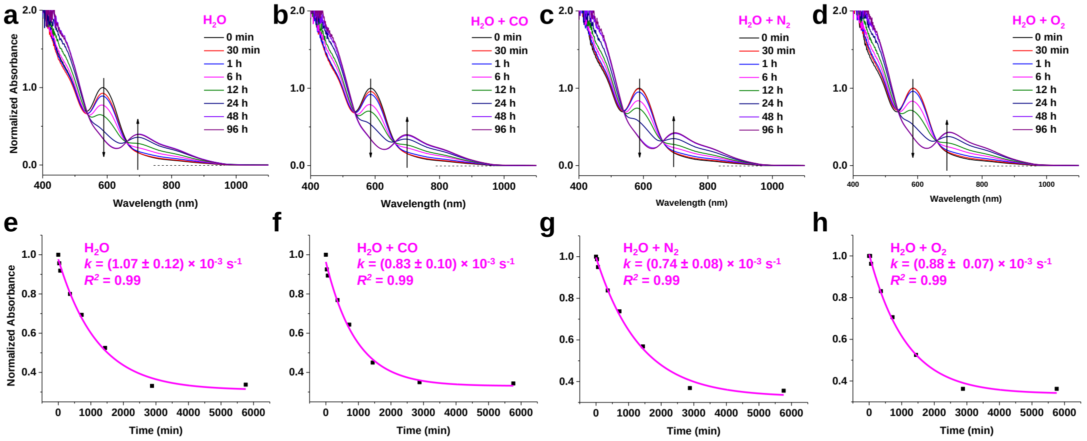

Supplementary Figure 5. (**a-d**) Time-course ultraviolet-visible absorption spectra and (**e-h**) reaction kinetic analyses of size conversion reaction from $[Au_{23}(SR)_{16}]^-$ to $[Au_{25}(SR)_{18}]^-$ in water saturated by different gases, where SR denotes thiolate ligand. Time-course absorption spectra in individual kinetic analysis are normalized to optical density at 589 nm at $t = 0$. The rate constant (k) is deduced according to the characteristic absorption decay profile of $[Au_{23}(SR)_{16}]^-$ by a pseudo-1st order reaction equation $OD_{@589} = OD_{@589,0} \times e^{-kt} + b$, where $OD_{@589,0}$ and $OD_{@589}$ are normalized initial and time-dependent optical density at 589 nm, respectively, while b is a constant accounting for the nonzero absorbance at 589 nm at the end state of reaction.

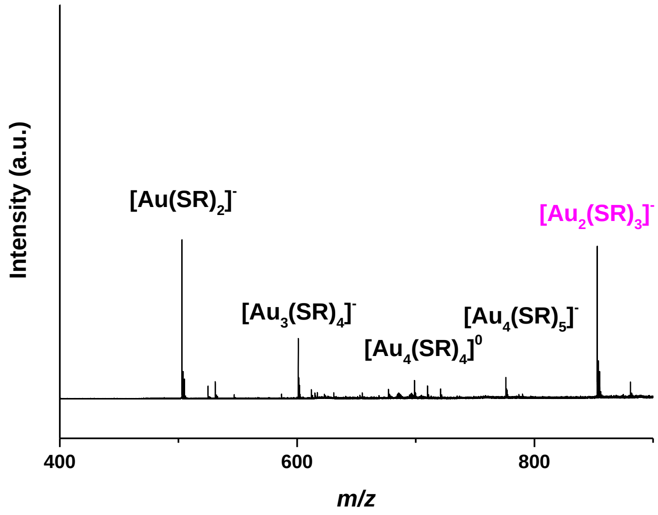

Supplementary Figure 6. Electrospray ionization mass spectrum of the ultrafiltrated $[Au_{23}(SR)_{16}]^-$ redissolved in water at low *m/z* end, where SR denotes thiolate ligand. The species extensively involved in the size conversion reaction is highlighted in magenta.

Supplementary Note 2. Kinetic analyses of size conversion reaction at varied ionic conditions

The ionic strength (I) could be estimated by the equation below:

$$I = \frac{1}{2}\sum_i c_i z_i^2 \tag{1}$$

where c_i and z_i denote concentration and charge number of ionic species i, respectively. We manipulated the ionic strength of the reaction solution by introducing different amounts of salt (e.g., NaCl) in solution, and conducted the size conversion reaction accordingly (Supplementary Fig. 7). In stark contrast to the reducing/oxidizing power of the reaction solution, the elevating ionic strength could significantly accelerate the size conversion kinetics (Fig. 2c, entries 5-8). Recent achievements in growth mechanism and structure determination of Au NCs revealed that halogen ligands possessed comparable bonding affinity to Au against thiolate ligands[2]. To fully rule out probable competing coordination of Cl induced size conversion, we also monitored the size conversion reaction in aqueous solutions containing altered anions (Supplementary Fig. 8). The resultant k value plots (Fig. 2c, entries 9-11) suggest that the reaction kinetics is rarely dependent on the chemical identities of anions, but it is highly related to the ionic strength.

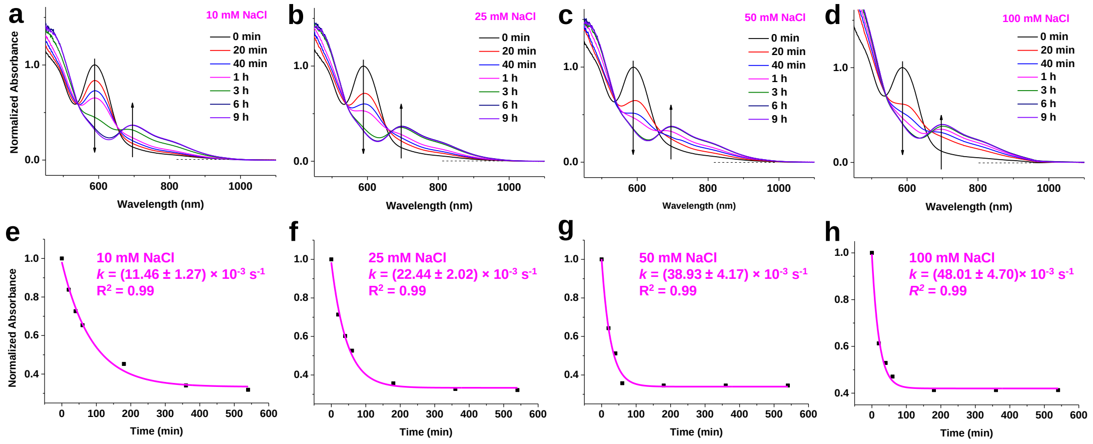

Supplementary Figure 7. (**a-d**) Time-course ultraviolet-visible absorption spectra and (**e-h**) reaction kinetic analyses of size conversion reaction from $[Au_{23}(SR)_{16}]^-$ to $[Au_{25}(SR)_{18}]^-$ in water at varied concentration of NaCl, where SR denotes thiolate ligand. Time-course absorption spectra in individual kinetic analysis are normalized to optical density at 589 nm at $t = 0$. The rate constant (k) is deduced according to the characteristic absorption decay profile of $[Au_{23}(SR)_{16}]^-$ by a pseudo-1st order reaction equation $OD_{@589} = OD_{@589,0} \times e^{-kt} + b$, where $OD_{@589,0}$ and $OD_{@589}$ are normalized initial and time-dependent optical density at 589 nm, respectively, while b is a constant accounting for the nonzero absorbance at 589 nm at the end state of reaction.

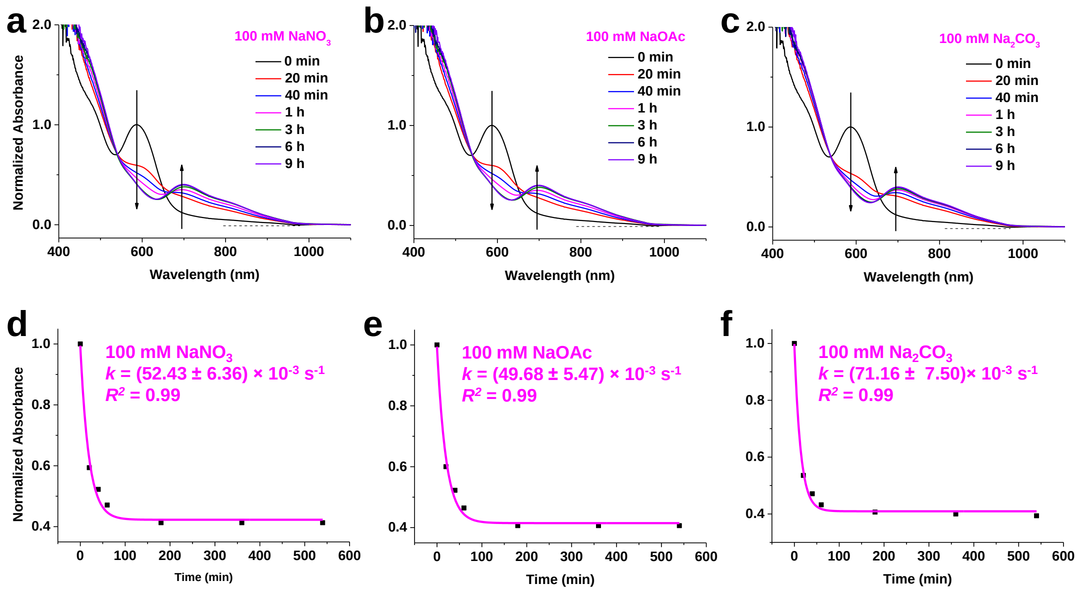

Supplementary Figure 8. (**a**-**c**) Time-course ultraviolet-visible absorption spectra and (**d**-**f**) reaction kinetic analyses of size conversion reaction from $[Au_{23}(SR)_{16}]^-$ to $[Au_{25}(SR)_{18}]^-$ in aqueous solutions of varied salts. Time-course absorption spectra in individual kinetic analysis are normalized to optical density at 589 nm at t = 0. The rate constant (k) is deduced according to the characteristic absorption decay profile of $[Au_{23}(SR)_{16}]^-$ by a pseudo-1st order reaction equation $OD_{@589} = OD_{@589,0} \times e^{-kt} + b$, where $OD_{@589,0}$ and $OD_{@589}$ are normalized initial and time-dependent optical density at 589 nm, respectively, while b is a constant accounting for the nonzero absorbance at 589 nm at the end state of reaction.

Supplementary Note 3. SR-[Au(I)-SR]$_2$ based surface-motif-exchange reaction

The ESI mass spectra reported in Fig. 3 suggest a SR-[Au(I)-SR]$_2$ based surface-motif-exchange (SME)-induced size conversion reaction. Such mechanism could first be understood by comparing the most intensive isotope peaks. As shown in Fig. 3i-3l, positive mass shifts of initially 6 Da (= 2 × 3 Da, Fig. 3j) and subsequently 3 Da (Fig. 3k and 3l) from the reference $[Au_{25}(p\text{-MBA})_{18}]^-$ peak (*p*-MBA = *para*-mercaptobenzoic acid) could be observed with increasing dosage of Au(I)-(*p*-NTP) complexes (*p*-NTP = *para*-nitrothiophenol). Given the molecular weight difference of the incoming *p*-NTP and *p*-MBA ligands to be 1 Da, such 3 Da based mass shift suggests the size conversion occurs by association of (*p*-NTP)-[Au-(*p*-NTP)]$_2$ to $[Au_{23}(p\text{-MBA})_{16}]^-$. Taking the centrosymmetry of $[Au_{23}(SR)_{16}]^-$ into account, the initial 6 Da mass shift suggests the dominant size conversion pathway as Supplementary Equation 2 below.

$$[Au_{23}(p\text{-MBA})_{16}]^- + 2\,[Au_2(p\text{-NTP})_3]^- \rightarrow [Au_{25}(p\text{-MBA})_{12}(p\text{-NTP})_6]^- + 2\,[Au(p\text{-MBA})_2]^- \quad (2)$$

In order to fully understand the size conversion reaction at molecular level, a minor discrepancy between experimental and simulated isotope patterns in Fig. 3i-3l should not be neglected. For example, three extra isotope peaks could be identified prior to the simulated isotope pattern of $[Au_{25}(p\text{-MBA})_{12}(p\text{-NTP})_6 - 3\,H]^{4-}$ in the experimental spectrum of Fig. 3j. Such extra peaks (Peaks 1-3 in Supplementary Fig. 9) could nevertheless be assigned to a side product of $[Au_{25}(p\text{-MBA})_{15}(p\text{-NTP})_3 - 3\,H]^{4-}$ (blue lines in Supplementary Fig. 9). More importantly, the perfect intensity profile match between such experimental (Peaks 1-3 in Supplementary Fig. 9) and simulated (blue lines in Supplementary Fig. 9) peaks rules out any possible presence of $[Au_{25}(p\text{-MBA})_{18\text{-}x}(p\text{-}$

NTP)$_x$]$^-$ (x = 1 or 2) species, agreeing perfectly with the proposed SR-[Au(I)-SR]$_2$ based SME mechanism. It should be reminded that the size conversion from [Au_{23}(p-MBA)$_{16}$]$^-$ to [Au_{25}(p-MBA)$_{18}$]$^-$ could be induced by the residual Au(I)-(p-MBA) complexes in the absence of the foreign Au(I)-(p-NTP) complexes (Fig. 1). Substituting one molecule of [Au_2(p-NTP)$_3$]$^-$ by the residual [Au_2(p-MBA)$_3$]$^-$ in Supplementary Equation 2 could yield [Au_{25}(p-MBA)$_{15}$(p-NTP)$_3$ – 3 H]$^{4-}$. Similarly, the experimental isotope patterns in Fig. 3k and 3l could be perfectly deconvoluted to [Au_{25}(p-MBA)$_{18-x}$(p-NTP)$_x$]$^-$ (x = 6, 9, and 12; Supplementary Fig. 9), where [Au_{25}(p-MBA)$_9$(p-NTP)$_9$]$^-$ and [Au_{25}(p-MBA)$_6$(p-NTP)$_{12}$]$^-$ are dominant products in the SME reactions with 0.5 and 1.0 mM Au(I)-(p-NTP) complexes, respectively. The as-evidenced stepwise mass increment by 3 Da unambiguously indicates the SR-[Au(I)-SR]$_2$ motif as the structure basis for the size conversion reaction.

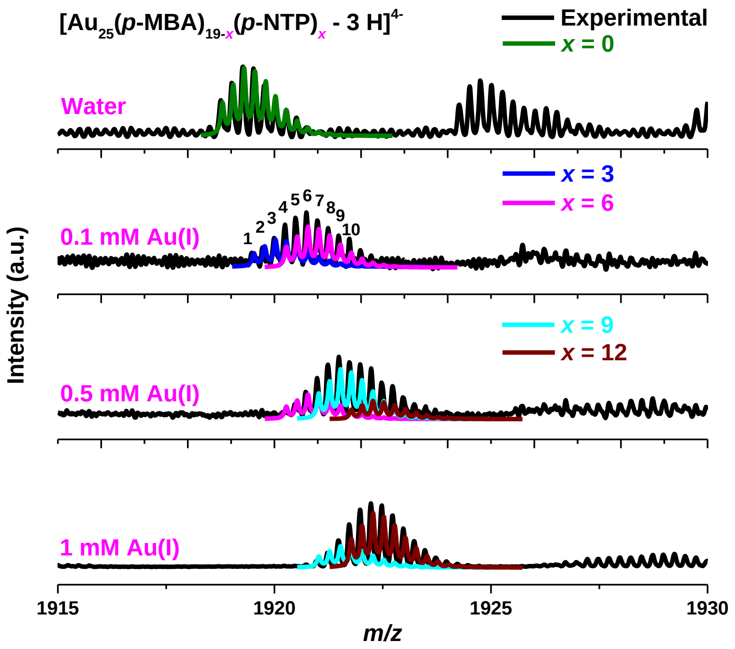

Supplementary Figure 9. Experimental (black lines) and simulated (colored lines) isotope patterns of $[Au_{25}(p\text{-MBA})_{18-x}(p\text{-NTP})_x]^-$ nanoclusters formed by reacting $[Au_{23}(p\text{-MBA})_{16}]^-$ with varied dosage of Au(I)-(*p*-NTP) complexes (expressed by [Au(I)] in each mass spectrum), where *p*-MBA and *p*-NTP are *para*-mercaptobenzoic acid and *para*-nitrothiophenol, respectively. Color code: olive, $x = 0$; blue, $x = 3$; magenta, $x = 6$; cyan, $x = 9$; wine, $x = 12$.

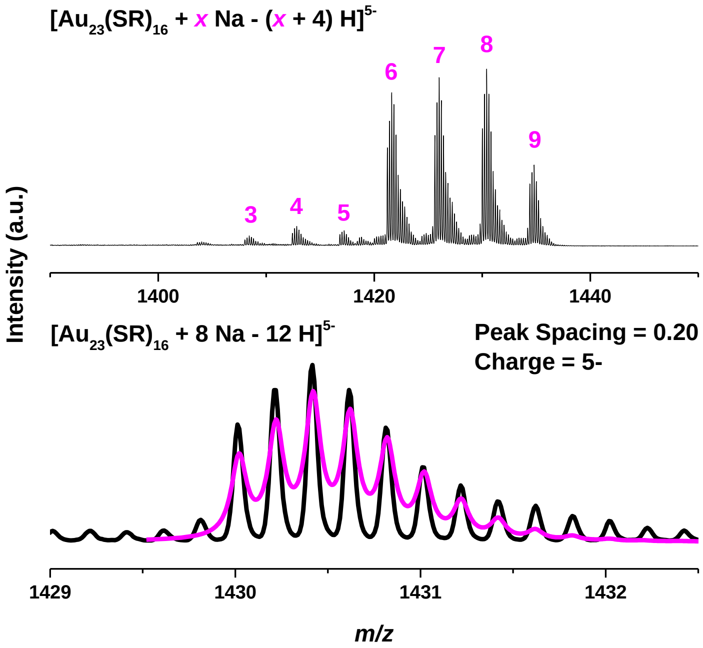

Supplementary Figure 10. Zoom-in electrospray ionization mass spectrum (top) and isotope patterns (bottom; black: experimental, magenta: simulated) of $[Au_{23}(SR)_{16} + x\ Na - (x + 4)\ H]^{5-}$ ions obtained in tandem mass spectrometry analysis. SR denotes thiolate ligand.

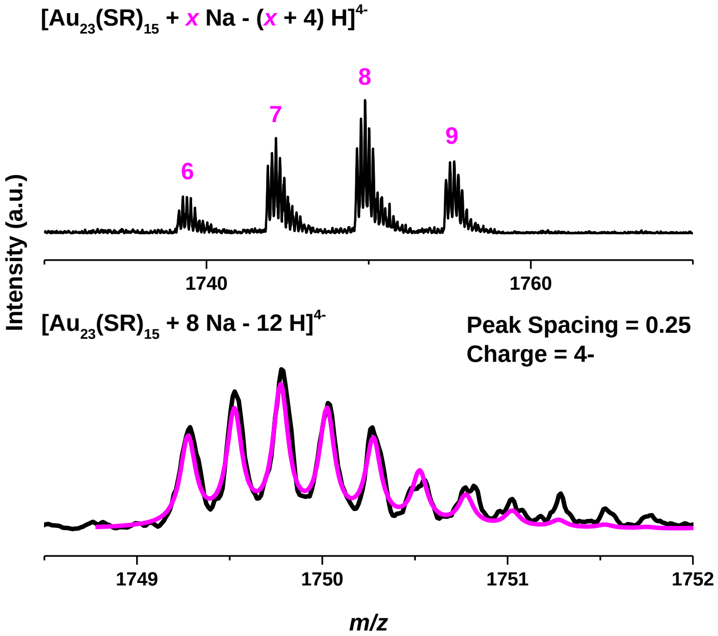

Supplementary Figure 11. Zoom-in electrospray ionization mass spectrum (top) and isotope patterns (bottom; black: experimental, magenta: simulated) of $[Au_{23}(SR)_{15} + x\ Na - (x + 4)\ H]^{4-}$ ions obtained in tandem mass spectrometry analysis. SR denotes thiolate ligand.

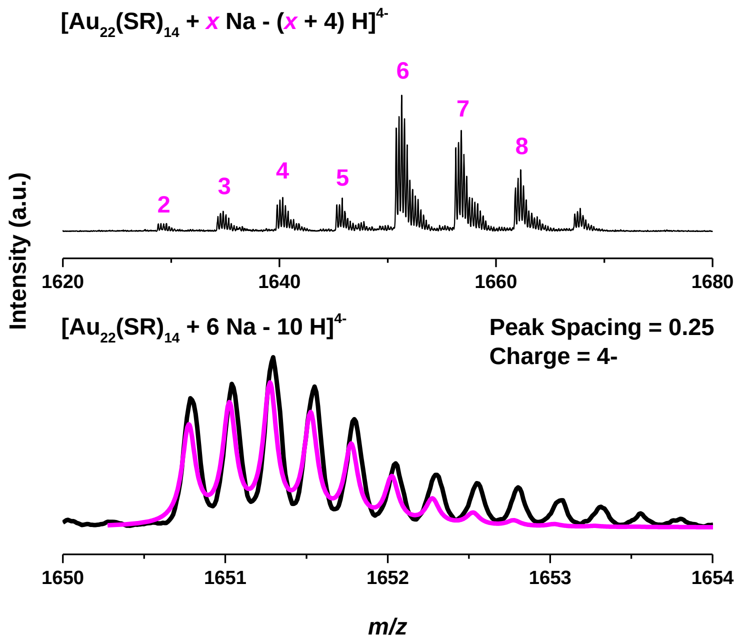

Supplementary Figure 12. Zoom-in electrospray ionization mass spectrum (top) and isotope patterns (bottom; black: experimental, magenta: simulated) of $[Au_{22}(SR)_{14} + x\ Na - (x + 4)\ H]^{4-}$ ions obtained in tandem mass spectrometry analysis. SR denotes thiolate ligand.

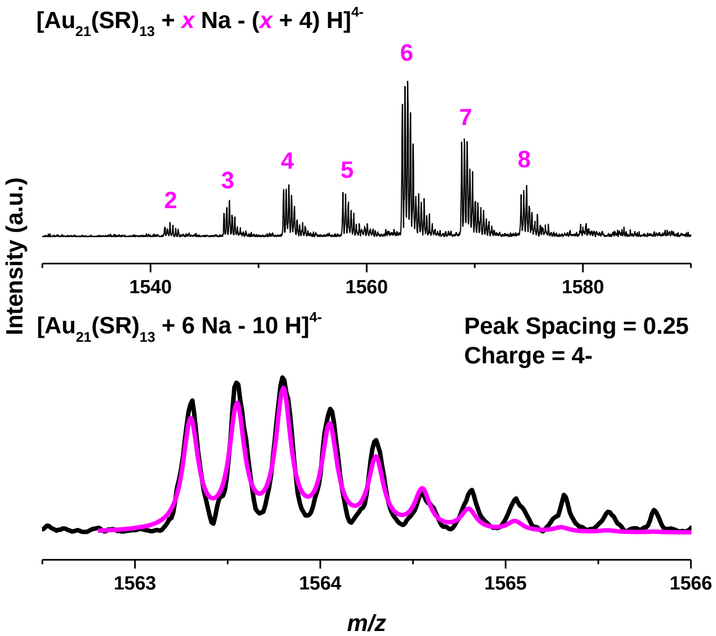

Supplementary Figure 13. Zoom-in electrospray ionization mass spectrum (top) and isotope patterns (bottom; black: experimental, magenta: simulated) of $[Au_{21}(SR)_{13} + x\ Na - (x + 4)\ H]^{4-}$ ions obtained in tandem mass spectrometry analysis. SR denotes thiolate ligand.

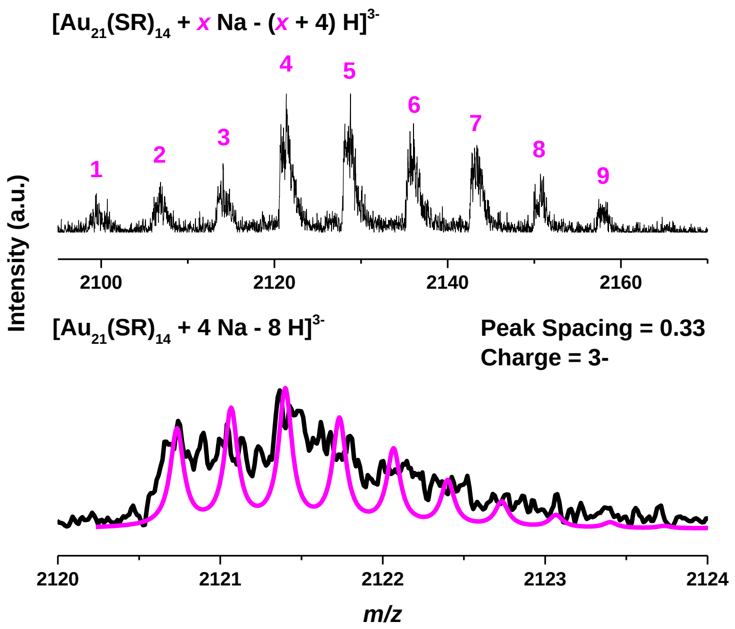

Supplementary Figure 14. Zoom-in electrospray ionization mass spectrum (top) and isotope patterns (bottom; black: experimental, magenta: simulated) of $[Au_{21}(SR)_{14} + x\ Na - (x + 4)\ H]^{3-}$ ions obtained in tandem mass spectrometry analysis. SR denotes thiolate ligand.

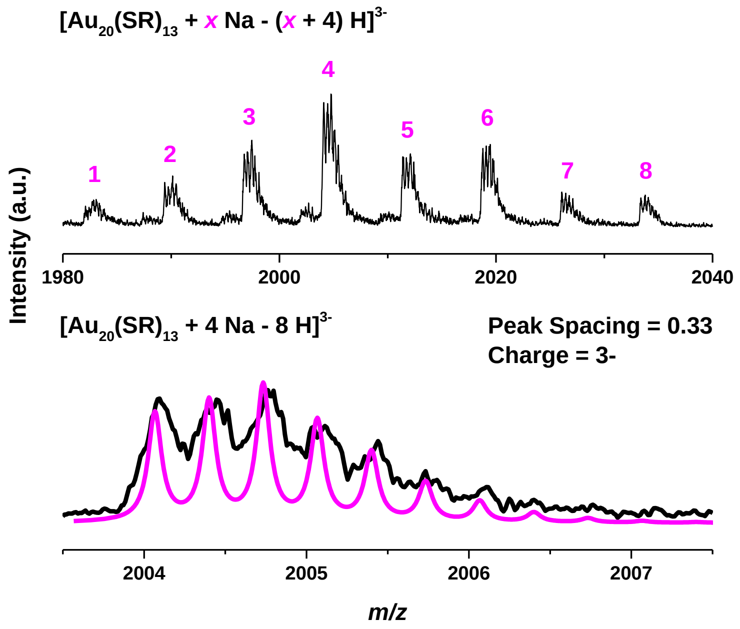

Supplementary Figure 15. Zoom-in electrospray ionization mass spectrum (top) and isotope patterns (bottom; black: experimental, magenta: simulated) of $[Au_{20}(SR)_{13} + x\ Na - (x + 4)\ H]^{3-}$ ions obtained in tandem mass spectrometry analysis. SR denotes thiolate ligand.

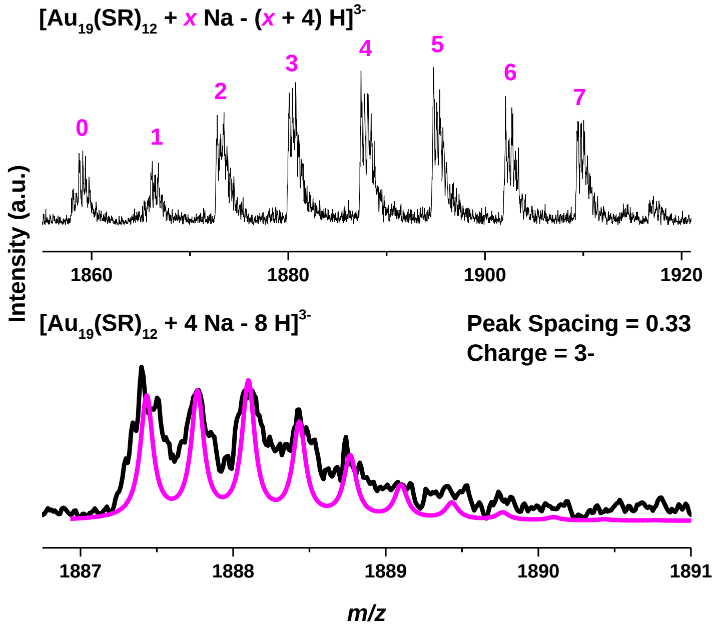

Supplementary Figure 16. Zoom-in electrospray ionization mass spectrum (top) and isotope patterns (bottom; black: experimental, magenta: simulated) of $[Au_{19}(SR)_{12} + x\ Na - (x + 4)\ H]^{3-}$ ions obtained in tandem mass spectrometry analysis. SR denotes thiolate ligand.

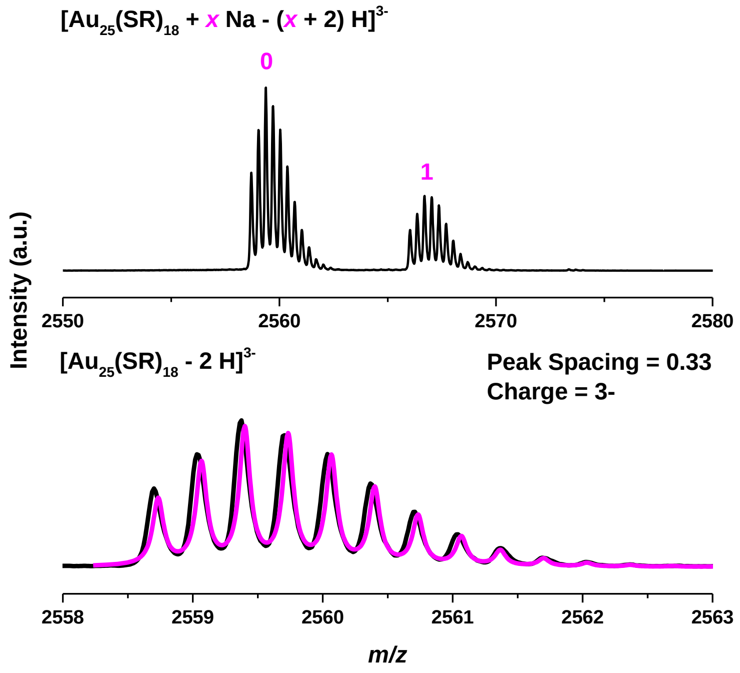

Supplementary Figure 17. Zoom-in electrospray ionization mass spectrum (top) and isotope patterns (bottom; black: experimental, magenta: simulated) of $[Au_{25}(SR)_{18} + x\ Na - (x + 2)\ H]^{3-}$ ions obtained in tandem mass spectrometry analysis. SR denotes thiolate ligand.

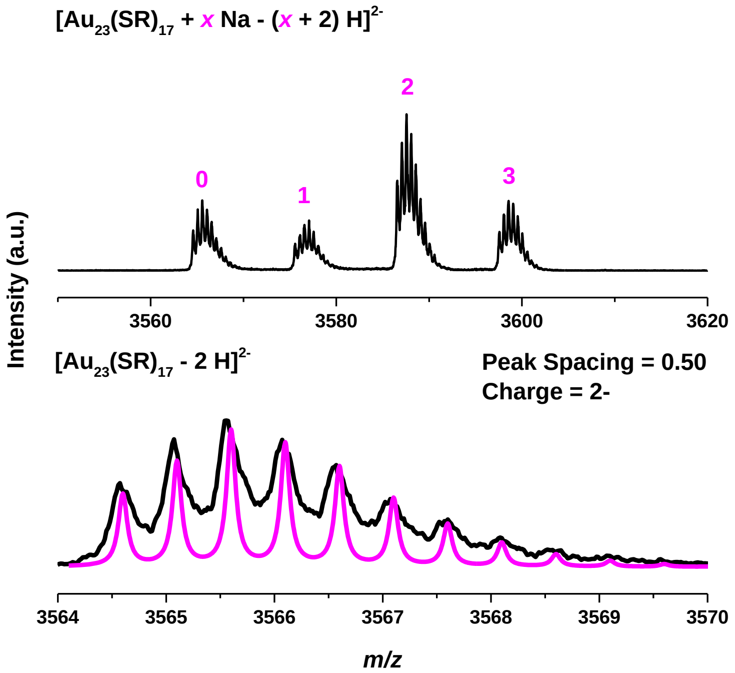

Supplementary Figure 18. Zoom-in electrospray ionization mass spectrum (top) and isotope patterns (bottom; black: experimental, magenta: simulated) of $[Au_{23}(SR)_{17} + x\ Na - (x + 2)\ H]^{2-}$ ions obtained in tandem mass spectrometry analysis. SR denotes thiolate ligand.

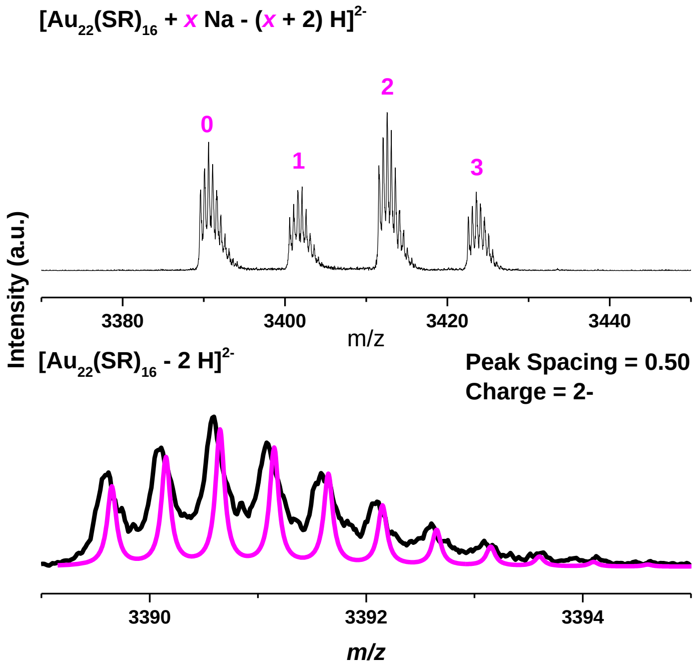

Supplementary Figure 19. Zoom-in electrospray ionization mass spectrum (top) and isotope patterns (bottom; black: experimental, magenta: simulated) of $[Au_{22}(SR)_{16} + x\ Na - (x + 2)\ H]^{2-}$ ions obtained in tandem mass spectrometry analysis. SR denotes thiolate ligand.

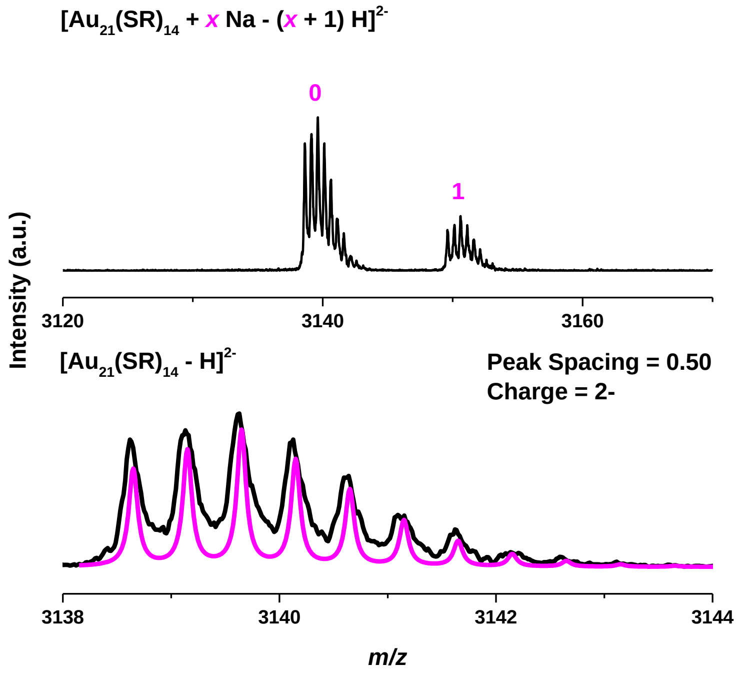

Supplementary Figure 20. Zoom-in electrospray ionization mass spectrum (top) and isotope patterns (bottom; black: experimental, magenta: simulated) of $[Au_{21}(SR)_{14} + x\ Na - (x + 1)\ H]^{2-}$ ions obtained in tandem mass spectrometry analysis. SR denotes thiolate ligand.

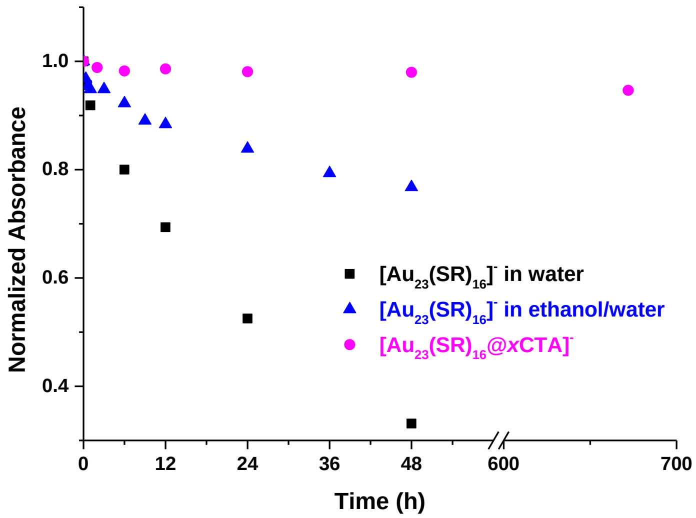

Supplementary Figure 21. Time-course absorbance of the characteristic peak at 589 nm of $[Au_{23}(SR)_{16}]^-$ and $[Au_{23}(SR)_{16}@x\text{CTA}]^-$ NCs, where SR and CTA denote thiolate and cetyltrimethylammonium ligands, respectively. $[Au_{23}(SR)_{16}]^-$ NCs are dissolved in water and the simulated mother liquid (water/ethanol = 6/4 Vol/Vol), while $[Au_{23}(SR)_{16}@x\text{CTA}]^-$ NCs are dissolved in ethanol. The original time-course ultraviolet-visible absorption spectra could be found in Fig. 2a, 6 and Supplementary Fig. 4, respectively. Time-course absorption spectra in individual kinetic analysis are normalized to optical density at 589 nm at $t = 0$.

Supplementary References

1. Yao, Q., Yuan, X., Yu, Y., Yu, Y., Xie, J. & Lee, J. Y. Introducing amphiphilicity to noble metal nanoclusters via phase-transfer driven ion-pairing reaction. *J. Am. Chem. Soc.* **137**, 2128-2136 (2015).

2. Yang, S., *et al.* A new crystal structure of Au_{36} with a Au_{14} kernel cocapped by thiolate and chloride. *J. Am. Chem. Soc.* **137**, 10033-10035 (2015).
